# Strontium inhibits titanium particle-induced osteoclast activation and chronic inflammation via suppression of NF-κB pathway

**DOI:** 10.1038/srep36251

**Published:** 2016-10-31

**Authors:** Shijun Zhu, Xuanyang Hu, Yunxia Tao, Zichuan Ping, Liangliang Wang, Jiawei Shi, Xiexing Wu, Wen Zhang, Huilin Yang, Zhikui Nie, Yaozeng Xu, Zhirong Wang, Dechun Geng

**Affiliations:** 1 Department of Orthopedics,The First Affiliated Hospital of Soochow University, 188, shi zi Road, Suzhou, 215006, China; 2 Department of Orthopedics, Jining No. 1 people’s hospital, 6, jiang kang Road, Jining, 272011, China; 3 Orthopedic Institute, Soochow University, 708, ren min Road, 215006, China; 4 Department of Orthopedics, Zhangjiagang Hospital of Traditional Chinese Medicine, 4, kang le Road, Zhangjiagang, 215600, China

## Abstract

Wear-particle-induced chronic inflammation and osteoclastogenesis have been identified as critical factors of aseptic loosening. Although strontium is known to be involved in osteoclast differentiation, its effect on particle-induced inflammatory osteolysis remains unclear. In this study, we investigate the potential impact and underling mechanism of strontium on particle-induced osteoclast activation and chronic inflammation *in vivo* and *in vitro*. As expected, strontium significantly inhibited titanium particle-induced inflammatory infiltration and prevented bone loss in a murine calvarial osteolysis model. Interestingly, the number of mature osteoclasts decreased after treatment with strontium *in vivo*, suggesting osteoclast formation might be inhibited by strontium. Additionally, low receptor activator of nuclear factor-κB ligand (RANKL), tumor necrosis factor-α, interleukin-1β, interleukin-6 and p65 immunochemistry staining were observed in strontium-treatment groups. *In vitro*, strontium obviously decreased osteoclast formation, osteoclastogenesis-related gene expression, osteoclastic bone resorption and pro-inflammatory cytokine expression in bone-marrow-derived macrophages in a dose-dependent manner. Furthermore, we demonstrated that strontium impaired osteoclastogenesis by blocking RANKL-induced activation of NF-κB pathway. In conclusion, our study demonstrated that strontium can significantly inhibit particle-induced osteoclast activation and inflammatory bone loss by disturbing the NF-κB pathway, and is an effective therapeutic agent for the treatment of wear particle-induced aseptic loosening.

Total joint arthroplasty is one of the most widely performed operations in recent years. However, it remains a time-limited solution for the damaged joint, since up to 30% of the patients can be revised within 20 years of the initial surgery^1^,^2^. The main reason of this pathological situation is peri-prosthetic osteolysis and subsequent aseptic loosening which has no satisfying therapeutic strategy. Wear particles released from the prosthesis are thought to play a central role in the initiation and development of osteolysis^3^,^4^.

Although the exact mechanism of aseptic loosening remains unclear, a key role has been attributed to inflammatory osteoclastogenesis in this process. Briefly, there are three important events in wear particle-induced-osteolysis, including chronic inflammation, osteoclast formation, and osteoclastic bone resorption^5^,^6^,^7^,^8^,^9^,^10^,^11^,^12^,^13^. Particles worn from prosthesis are easily phagocytized by the peri-prosthetic phagocytes in the tissue^4^. Engulfing these wear particles, phagocytes can be activated and secrete amount of pro-inflammatory cytokines, such as tumor necrosis factor-α (TNF-α), interleukin-1β (IL-1β) and IL-6^5^,^7^,^9^,^11^,^12^. These factors mediate fibroblasts and osteoblasts secreting high levels of receptor activator of nuclear factor-κB (RANK) ligand (RANKL)^14^,^15^. RANKL plays an activated role in osteoclastogenesis by connecting the corresponding receptor RANK on the membrane of osteoclast precursors^16^. Briefly, binding of RANKL to RANK initiates the recruitment of TNF receptor-associated factor 6 and prompts induction of the nuclear factor-κB (NF-κB) pathway which induces osteoclastogenesis finally to result in the peri-prosthetic osteolysis^17^,^18^,^19^.

Strontium, a trace element chemically close to calcium, induces pharmacological actions on bone metabolism. A compound containing two atoms of strontium (strontium ranelate, SrRan) was found to have a therapeutic effect on osteoporosis by postmenopausal woman^20^. *In vitro* data have demonstrated that strontium acts in part by decreasing osteoclast formation and bone resorption^21^,^22^,^23^,^24^. It also reduces the differentiation of osteoclasts by modulating the NF-κB pathway, as well as the number and activity of osteoclasts^23^,^24^. In addition, strontium also inhibits the expression of pro-inflammatory cytokines, such as TNF-α and IL-6^25^. Since these factors play an important role in aseptic loosening, it’s reasonable to believe that strontium might be a good agent for treating aseptic loosening. We and Lu *et al*. have demonstrated that SrRan impaired particle-stimulated bone destruction in a murine calvarial model^26^,^27^. However, the precise mechanism of strontium on wear-debris-induced inflammatory osteolysis remains unclear.

Here, we investigated the potential impact of strontium on titanium particle-induced osteolysis *in vivo* and *in vitro*. And our data showed that strontium obviously inhibited titanium particle-induced inflammatory bone destruction and osteoclatogenesis through the suppression of NF-κB pathway. The results of this study provide a possible mechanistic explanation for the protective effect of strontium against wear-debris-induced osteolysis and provide a rationale for strontium use in the treatment of peri-prosthetic osteolysis and subsequent aseptic loosening.

## Results

### Strontium ranelate reduces titanium particle-induced inflammatory osteolysis and prevents bone loss *in vivo*

**Figure 1:**
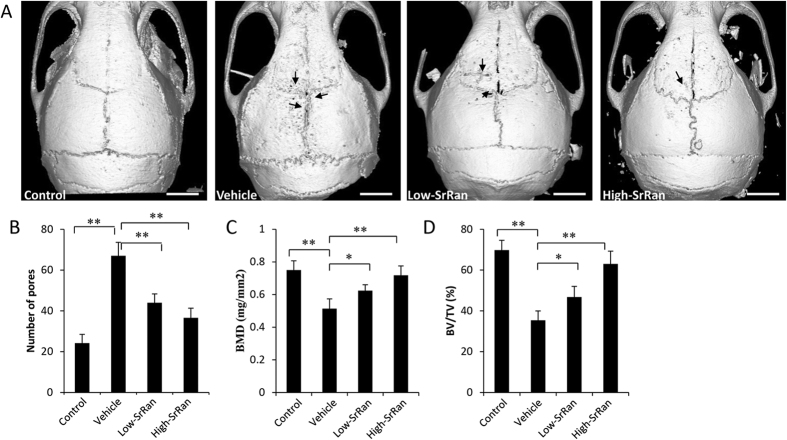
SrRan inhibits bone destruction in murine calvariae model. (**A**) Representative μCT images (Scar bar, 5 mm), (**B**) the number of pores, (**C**) BMD and (**D**) BV/TV of each mice within ROI were measured. *P < 0.05 and **P < 0.01.

We established a mouse calvarial osteolysis model to investigate the effect of SrRan on Ti particle-induced bone loss. μCT results showed that extensive bone resorption mainly occurred in the calvaria of Ti particle-stimulated mice. In SrRan-treatment groups, particle-induced osteolysis was reduced in a dose-dependent manner, and bone resorption in mice treated with the high-SrRan concentration was much less than in mice treated with the low-SrRan concentration (Fig. 1A). Quantification of bone parameters revealed that Ti particle stimulation results an obvious increase in the number of pores, and a decrease in BMD and BV/TV in the calvarial ROI’s (*P* < 0.05). In contrast, when SrRan was given at 450 mg/kg and 900 mg/kg daily, particle-induced bone loss was prevented compared to the vehicle group (*P* < 0.05; Fig. 1B–D).

**Figure 2:**
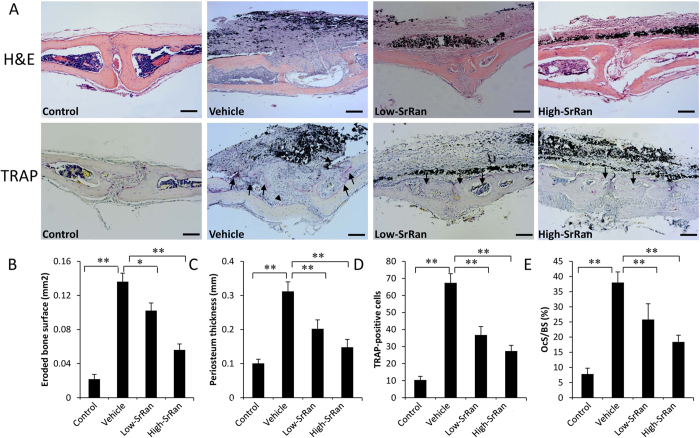
SrRan suppresses titanium particle-induced inflammatory response and osteoclast formation *in vivo*. (**A**) Representative slices of hematoxylin and eosin (H&E) and tartrate-resistant acid phosphatase (TRAP) staining (Scar bar, 100 μm). (**B**) Histomorphometric analysis of the area of eroded bone surface, (**C**) periosteum thickness, (**D**) number of mature osteoclasts and (**E**) the percentage of osteoclast per bone surface within the ROI in each group were measured. *P < 0.05 and **P < 0.01.

The histological staining and histomorphometric analysis further confirmed that SrRan treatment attenuates particle-induced bone loss *in vivo*. H&E results revealed that an obvious inflammatory infiltration of lymphocytes and macrophages, as well as multinucleated osteoclasts, were present in the calvariae of particle-stimulated mice. TRAP staining showed lots of TRAP-positive cells accumulated along the eroded bone surface in particle-stimulated mice (Fig. 2A). Consistent with the μCT quantification, histomorphometric results showed that daily gavage-fed SrRan at 450 mg/kg and 900 mg/kg obviously decreased the area of eroded bone surface and periosteum thickness induced by Ti particles (*P* < 0.05; Fig. 2B,C). In addition, the number of TRAP-positive cells and the percentage of OCs/BS were reduced in both the low- and high-concentration SrRan groups (*P* < 0.05; Fig. 2D,E).

### Strontium ranelate and the expression of RANKL, TNF-α, IL-1β and IL-6

**Figure 3:**
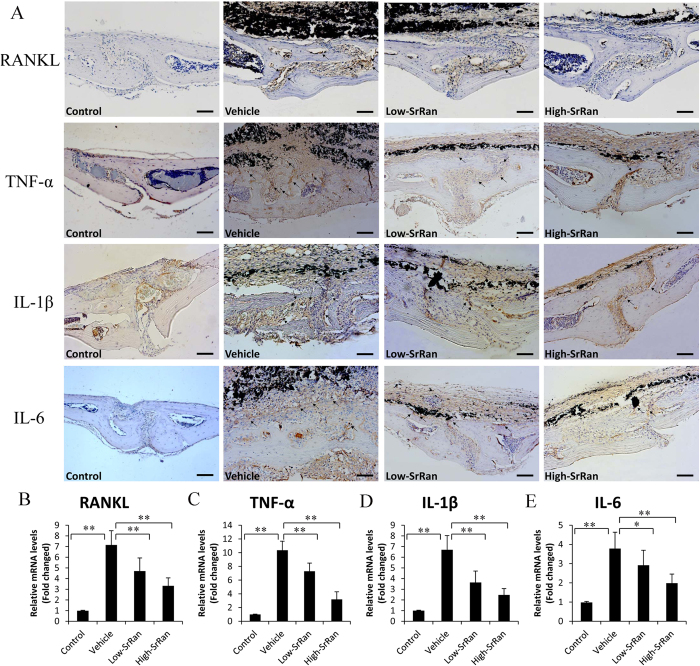
Expression of RANKL, TNF-α, IL-1β and IL-6 in mouse calvariae. (**A**) Immunohistochemical staining for RANKL, TNF-α, IL-1β and IL-6 in mouse calvariae of each group (Scar bar, 100 μm). (**B**) The mRNA levels of RANKL, TNF-α, IL-1β and IL-6 were determined by RT-PCR. *P < 0.05 and **P < 0.01.

After establishing that SrRan reduced mature osteoclast numbers in osteolytic sites stimulated with Ti particles, and our previous studies have demonstrated that RANKL is critical in mediating wear-debris induced osteoclastogenesis^7^,^9^,^13^, we want to determine whether SrRan could modify the expression of RANKL in calvaria of particle-stimulated mice. As shown in Fig. 3A, a large overexpression of RANKL was observed at the surface of eroded bone in particle-stimulated mice compared with a low expression in vehicle group. Meanwhile, RT-PCR showed that Ti particle significantly increased the expression of RANKL gene transcripts in calvarial of mice compared to the vehicle group (*P* < 0.05). In contrast, SrRan treatment significantly decreased the expression of RANKL even in the presence of Ti particles (*P* < 0.05; Fig. 3B).

Histological assessment and histomorphometric analysis demonstrated that SrRan treatment inhibited particle-stimulated inflammatory reaction characterized by the thickness of inflammatory periosteum infiltrated by lymphocytes and macrophages compared with vehicle group, we further explored the expression pro-inflammatory cytokines *in vivo*. As expected, intense TNF-α, IL-1β and IL-6 staining were observed in calvaria of particle-stimulated mice. However, the elevation of these cytokines was significantly reduced in both the Low- and High-concentration SrRan treatment mice. The gene levels of TNF-α, IL-1β and IL-6 determined by RT-PCR were shown in Fig. 3C–E, Ti particles significantly increased TNF-α, IL-1β and IL-6 gene expression in calvaria compared with vehicle group (*P* < 0.05). However the number of gene copies of TNF-α, IL-1β and IL-6 was significantly reduced by SrRan treatment (*P* < 0.05).

### Strontium and RANKL-induced osteoclast formation

**Figure 4:**
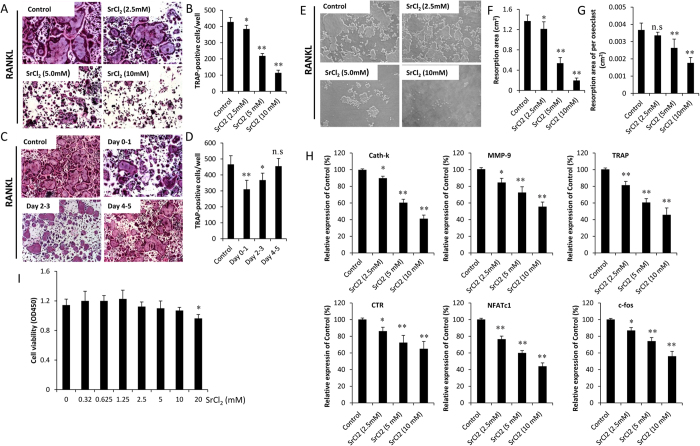
SrCl_2_ impairs RANKL-induced osteoclast formation and osteoclast marker gene expression. (**A**) BMMs were treated with various concentration of SrCl_2_ and then cultured with induction medium for 5 days. TRAP staining was performed to identify mature osteoclast. (**B**) The numbers of TRAP-positive multinucleated cells were determined. (**C**) BMMs were incubated with induction medium and exposed to 5 mM SrCl_2_ on days 0–1, days 2–3 and days 4–5. The TRAP staining was performed at Day 5. (**D**) The numbers of TRAP-positive multinucleated cells were determined. (**E**) BMMs were cultured with 30 ng/ml M-CSF, 50 ng/ml RANKL, 0.1 mg/ml Ti particles and various concentration of SrCl2 (2.5, 5 and 10 mM) on Osteo Assay Plate for 5 days. Bone resorption lacuna was observed under microscopy. (**F**) Resorption area and (**G**) resorption area per osteoclast were determined. (**H**) SrCl_2_ inhibits RANKL-induced genes expression of *Cath-k, MMP-9, TRAP, CTR, NFATc1 and c-fos*. (**I**) BMMs were incubated with induction medium and various concentration of SrCl_2_ for 48 h. Cell viability was determined by using CCK-8 assay. All experiments were performed at least three times. n.s: no significant; *P <  0.05 and **P <  0.01 compared with control group.

We then investigated the effect of strontium on RANKL-induced osteoclast formation *in vitro*. BMMs were treated with/without various concentration of SrCl_2_ in the presence of 30 ng/ml M-CSF, 50 ng/ml RANKL and 0.1 mg/ml Ti particles. Numerous TRAP-positive cells were observed in the group without SrCl_2_ treatment (control group). In contrast, SrCl_2_ treatment markedly reduced the number of TRAP-positive cells in a dose-dependent manner (*P* < 0.05; Fig. 4A,B). To determine which stages of osteoclast formation were affected, BMMs were exposed to 5 mM SrCl_2_ for 1 day. The TRAP staining was performed at Day 5. The results showed that the addition of SrCl_2_ on days 0–1 and days 2–3 markedly reduced TRAP-positive cell formation (*P* < 0.05). However, SrCl_2_ treatment at later stage (Days 4–5) did not effectively attenuate osteoclast differentiation (*P* > 0.05; Fig. 4C,D). These findings indicate that strontium impaired RANKL-induced osteoclast formation in the early stages rather than late stages. We then examined the inhibitory effect of strontium on bone resorption using the OAP plates. The result showed that strontium could significantly inhibit RANKL-induced bone resorption. In addition, the resorption activity of individual osteoclasts was also affected by strontium (*P* < 0.05; Fig. 4E–G). To exclude the possibility that the inhibitory effects of strontium on osteoclast formation was due to cytotoxic effects of strontium, a CCK-8 assay was used to test the viability of BMMs treated with strontium. As shown in Fig. 4I, the viability of cells cultured with different concentration of strontium was not significantly decreased at each time point compared with the control group (*P* > 0.05).

### Effects of strontium on RANKL-induced gene expression

RT-PCR demonstrated that the RANKL-induced mRNA levels of osteoclast-related genes, including *Cath-K, CTR, MMP-9, NFATc1, c-fos* and *TRAP* were significantly increased after stimulated with RANKL for 5 days (*P* < 0.05). When the cells were treated with SrCl_2_, the increase of *Cath-K, CTR, MMP-9, NFATc1, c-fos* and *TRAP* mRNA levels were obviously suppressed (*P* < 0.05; Fig. 4H).

### Effects of strontium on pro-inflammatory cytokine expression

**Figure 5:**
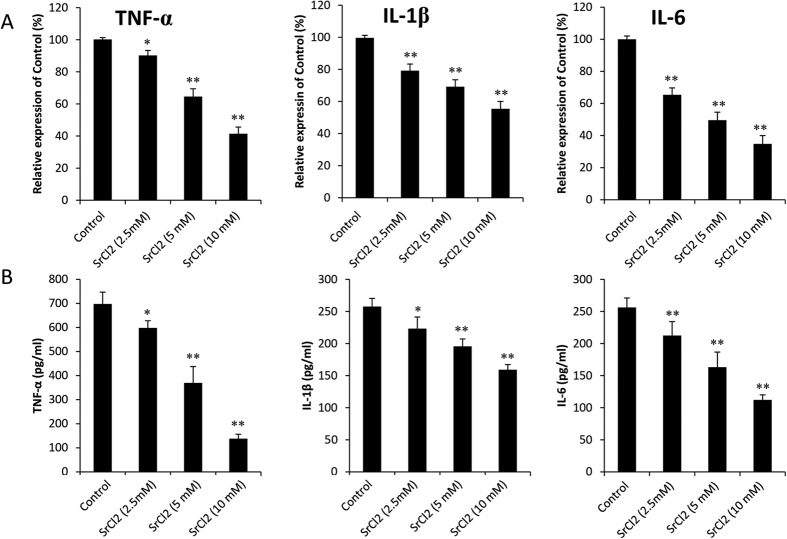
SrCl_2_ suppressed the expression of TNF-α, IL-1β and IL-6 *in vitro*. BMMs were cultured with 30 ng/ml M-CSF, 50 ng/ml RANKL, 0.1 mg/ml Ti particles and various concentration of SrCl_2_ (2.5, 5 and 10 mM) for 24 hours. (**A**) The gene copies of *TNF-α, IL-1β* and *IL-6* were determined by using RT-PCR. (**B**) The protein expression of those inflammatory cytokines in the supernatant was analyzed by ELISA. All experiments were performed at least three times. *P <  0.05 and **P <  0.01 compared with control group.

As shown in Fig. 5A, the mRNA levels of *TNF-α, IL-1β*, and *IL-6* were induced in control group. In contrast, the expression of those inflammation-related genes was markedly suppressed by strontium treatment (*P* < 0.05). To assess whether SrCl_2_ can inhibit the secretion of pro-inflammatory cytokines that are stimulated by Ti particles, we examined the concentration of TNF-α, IL-1β, and IL-6 in supernatant cultured with BMMs using ELISA assays. The presence of Ti particles induced a significant increase in the protein levels of TNF-α (630.7 ± 64.9) pg/ml, IL-1β (301.4 ± 31.9)pg/ml and IL-6 (243.6 ± 36.7)pg/ml. When SrCl_2_ was added to the culture system, the protein levels of these cytokines were obviously reduced (*P* < 0.05; Fig. 5B).

### Strontium and NF-κB signaling pathway

**Figure 6:**
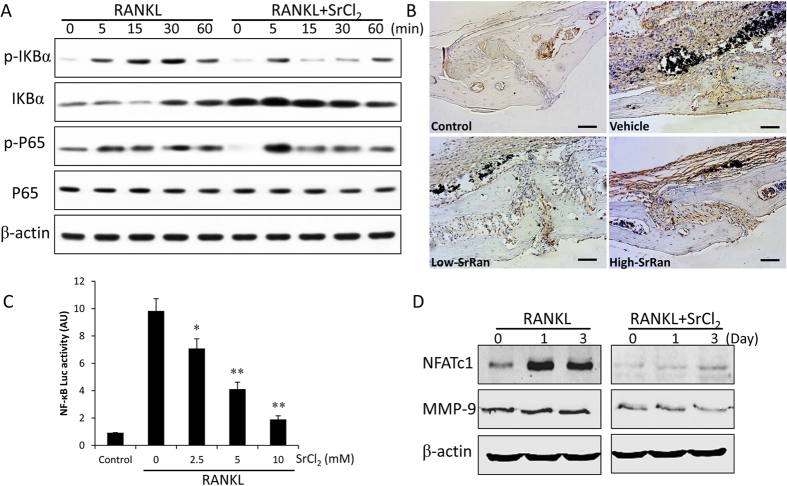
Strontium attenuates the activation of NF-κB pathway. (**A**) RAW264.7 cells were incubated with 50 ng/ml RANKL, 0.1 mg/ml Ti particles with or without 5 mM SrCl_2_ for indicated times. Cells were lysed for western blotting with specific antibodies against phosphor-IκBα, IκBα, phosphor-p65, p65 and β-actin. (**B**) Immunohistochemical staining for p65 in mouse calvariae of each group. (**C**) RAW264.7 cells transiently transfected with NF-κB-Luc reporter plasmids and pretreated with or without SrCl_2_ (5 mM) for 1 h, followed by incubation with RANKL and Ti particles for 6 h. Transcriptional activity was evaluated by luciferase assay. (**D**) BMMs were pretreated with or without SrCl_2_ (5 mM) in the presence of RANKL and Ti particles for indicated times, and NFATc1 and MMP-9 protein levels were analyzed by western blot. All experiments were performed at least three times. *P < 0.05 and **P < 0.01 compared with RANKL alone group.

To investigate the molecular mechanism underlying the inhibitory effect of strontium on wear particle-induced osteoclastogenesis, we performed western blot assay. After pre-treatment with SrCl_2_ (5 mM) for 4 h, RAW264.7 cells were stimulated by 50 ng/ml RANKL for indicated time (0, 5, 15, 30, 60 min). As shown in Fig. 6A, RANKL obviously induced phosphorylation and degradation of IkBa after 15 min. However, SrCl_2_ pretreatment significantly inhibited RANKL-induced IkBa phosphorylation and degradation in RAW264.7 cells. In addition, p65 phosphorylation was also significantly diminished by SrCl_2_. Meanwhile, the immuohistochemical staining reveals that SrCl_2_ suppressed the expression of p65 in a dose-dependent manner at osteolytic sites stimulated by Ti particles (Fig. 6B). In accordance with western blot and immuohistochemical results, Ti particle was able to induce more than 9.8 fold of luciferase activity in RAW264.7 cells transfected with NF-κB-luc after 6 h of treatment. Interestingly, the luciferase activity was significant reduced by SrCl_2_ even in response to Ti particles (*P* < 0.05; Fig. 6C). To determine whether SrCl_2_ inhibits NF-κB dependent transcription in BMMs, protein levels of NF-κB two target factors, NFATc1 and MMP-9 which play significant role in osteoclast differentiation, were measured by western blot after RANKL stimulation for various time. The results demonstrated that pretreatment of SrCl_2_ for 4 h significantly reduced RANKL-induced expression of NFATc1 and MMP-9 (Fig. 6D).

## Discussion

SrRan, a novel anti-osteoporosis agent, has been reported to exert beneficial effects on osteoporotic bones in postmenopausal women and in ovariectomy-induced osteoporosis models^20^,^28^. At the cellular level, the beneficial effects are based on the dual effect of strontium on osteoblasts and osteoclasts^24^. Previous studies have demonstrated that strontium attenuated particle-induced bone destruction *in vivo*^26^,^27^. However, the precise mechanism remains unclear. The aim of this study was to improve the understanding of strontium as a therapeutic agent to prevent wear-debris-associated osteolysis in a murine calvarial model, and to investigate the potential mechanism of strontium on particle-induced inflammatory osteoclastogenesis.

The present study has demonstrated that strontium significantly increased bone volume and reduced osteolysis, which is consistent with previous studies^13^. These findings gave rise to the question as to the mechanism of strontium action in the prevention of wear-debris-induced osteolysis.

Activated osteoclastogenesis has been recognized as a common characteristic among various forms of bone loss disease, such as osteoporosis, cancer-associated bone disease and peri-prosthetic osteolysis. Infiltration of multinucleated osteoclast in model and peri-prosthesis tissue has been well defined, and our previous work confirmed that Ti particles significantly increased osteoclastogenesis *in vivo* and *in vitro*^8^,^9^,^12^,^13^. Therapeutic strategy targeting osteoclast formation significant inhibits particle-associated bone resorption^10^,^13^. Therefore, we considered whether the protective effects of strontium were mediated by the inhibition of osteoclast formation and osteoclatic bone resorption. As expect, in this study, strontium treatment significantly reduced osteoclast formation, and decreased resorption area, findings which consistent with previous reports^22^,^24^. In addition, we confirmed that strontium impaired osteoclast formation by evaluating the gene levels of *Cath-K, CTR, MMP-9, NFATc1, c-fos* and *TRAP*. Furthermore, in an *in vivo* experiment, strontium obviously decreased eroded bone surface and number of TRAP-positive cells when compared to vehicle group. These results indicated that strontium mediates its protective effect on particle-induced osteolysis through inhibiting on osteoclast formation and osteoclatic bone resorption.

RANKL is now recognized as a key regulator of osteoclast formation which regulates bone resorption in both healthy and diseased states^8^,^29^. Recent studies have showed that RANKL is overexpressed in tissues adjacent to failure implant^8^. Therapeutic methods which down-regulated RANKL expression have been shown to effectively inhibits particle-stimulated osteoclastic bone resorption^9^,^13^. Using the murine calvarial model, we observed that RANKL expression significantly increased in particle-stimulated mice when compared with the sham group. However, when the mice were treated with strontium for 2 weeks, the expression level of RANKL was significantly decreased, which is consistent with previous studies^26^. Brennan *et al*. demonstrated that strontium suppresses RANKL expression in osteoblasts via activation of calcium sensing receptor signaling pathway^30^. During aseptic loosening, RANKL is mainly expressed by fibroblasts and osteoblasts under the stimulation with pro-inflammatory cytokines, including TNF-α, IL-1β and IL-6^14^,^15^. In the current study, we observed that strontium inhibits those pro-inflammatory cytokines expression via modulation NF-κB pathway, and this may partially explain the down-regulation of RANKL in strontium-treatment groups. These data showed that the inhibitory effects of strontium on particle-induced bone destruction are mediated by suppression RANKL expression *in vivo*.

Particle-stimulated macrophages released a multitude of pro-inflammatory cytokine, including TNF-α, IL-1β, and IL-6. It is believed that the localized release of these substances can promote wear-particle-induced osteoclastogenesis and subsequent processes leading to osteolysis^4^,^5^,^6^. Since strontium is reported to have regulating effects on inflammatory disease^25^, we investigate the effects of Ti particles on them and the role of strontium in these events. Our data showed that strontium obviously decreased the expression of TNF-α, IL-1β, and IL-6, and subsequently suppressed inflammatory cell infiltration. These results indicated that strontium-mediated anti-inflammatory effects represent a major cause of the protective effects of strontium on wear-debris-induced osteolysis.

Since NF-κB is one of the central transcriptional factors in regulating the differentiation of osteoclasts^31^,^32^, we asked whether or not the action of strontium is mediated through inhibiting NF-κB activity. In this study, we found that Ti particle significantly promotes NF-κB activity in RAW264.7 cells. As expected, the particle-induced NF-κB activity was significantly inhibited by prior treatment with strontium. In addition, strontium also attenuates the protein levels of NFATc1 and MMP-9 as compared with untreated controls. Several authors have demonstrated that blocking NF-κB pathway obviously diminished wear-particle-induced osteoclastogenesis^18^,^19^. Similarly, our results showed that strontium significantly decreased particle-induced osteoclast formation and bone resorption. Recent studies have showed that the inhibitory effect of strontium on osteoclast activation is involved to NF-κB pathway^23^,^24^, findings that are consistent with our results. These results suggest that the inhibitory effect of strontium on particle-induced osteoclastogenesis is targeted against not only the NF-κB activity but also the down-stream factors of NF-κB pathway.

NF-κB is also a critical regulator of inflammatory signals^31^. It was reported that NF-κB up-regulated TNF-α and IL-1β expression by monocytes^32^. Meanwhile, previous studies demonstrated that NF-κB pathway was activated by wear particles and subsequently promoted pro-inflammatory cytokines expression^9^,^33^. In addition, blockade NF-κB activity results in a significant reduction in pro-inflammatory cytokines. In the current study, our results showed that strontium obviously decreased the protein level of TNF-α, IL-1β and IL-6 stimulated by Ti particles, as compared to that without Ti particles. Interestingly, strontium also significant inhibits particle-induced NF-κB activation. These results suggested that the impaction of strontium on inflammatory cytokines expression was mediated via the inhibition of NF-κB activation.

Collectively, the present study showed that strontium efficiently prevented particle-induced bone loss. We also demonstrated that the protective effects of strontium were mainly mediated through an inhibition of osteoclast formation and inflammatory responses via the down-regulation of NF-κB pathway. Hence, it is strongly suggested that strontium could be developed as a potential agent for the prevention and treatment of wear particle-induced osteolysis and subsequent aseptic loosening.

## Materials and Methods

The animal experiments were performed in strict accordance with the principles and procedures of the National Institutes of Health (NIH) Guide for the Care and Use of Laboratory Animals and the Animal Care Committee of the First Affiliated Hospital of Soochow University. All experimental protocols in this study were approved by the Ethics Committee of the First Affiliated Hospital of Soochow University.

### Reagents

Recombinant murine receptor activator of nuclear factor-κB ligand (RANKL) and macrophage colony stimulating factor (M-CSF) were purchased from R&D systems (Minneapolis, MN). The strontium chloride (SrCl_2_) was purchased from Sigma-Aldrich (St. Louis, MO, USA). SrRan was provided by Les Laboratories Servier Industrie (Gidy, France). Cell culture media Minimum Essential Medium, Alpha Modified (α-MEM) was obtained from GIBCO-BRL (Grand Island, NY, USA). The murine-specific ELISA kits were purchased from Biosource International Inc (Camarillo, California, USA). The murine macrophage cell line RAW264.7 was obtained from the American Type Culture Collection (Manassas, VA).

### Titanium particle preparation

Commercially titanium (Ti) particles were purchased from Johnson Matthey (Ward Hill, MA, USA). As previously described^7^,^9^,^13^, we removed the endotoxin attached on particles by sterilizing at 180 °C for 6 h and then washing with 75% ethanol solution for 48 h. The endotoxin level of the particles was lower than 0.1EU/ml, as determined by a Limulus assay kit used in accordance with the manufacturer’s instructions (QCL-1000; Biowhittaker, Walkersville, MD). We used a SB3200 Ultrasonic Generator (Shanghai Branson Ultrasonics, Shanghai, China) to blend the Ti particles suspension before culturing with cells. Based on the research of several experiments, this study chose the mass action concentration of Ti particles as 0.1 mg/ml, which could effectively mimic wear particles obtained from peri-prosthetic tissue^6^,^14^.

### Cell culture and osteoclast differentiation

Bone-marrow-macrophages (BMMs) were extracted from the bone marrow of 4- to 6-week-old male C57/BL6 mice. Briefly, cells were separated and cultured in α-MEM containing 10% FBS, 2 mM L-glutamine, 100 U/ml penicillin/streptomycin and M-CSF (30 ng/mL) for 24 h. Adherent cells were removed and the non-adherent cells were cultured in a 37 °C/5% CO_2_ incubator in 6-well plates for another 3 days until fully confluent. The BMMs were seeded into 96-well plates at a density of 1 × 10^4^ cells/well in α-MEM containing 10% FBS, 30 ng/mL M-CSF, 50 ng/mL RANKL, 0.1 mg/ml Ti particles with/without a various concentration of SrCl_2_. The medium was changed every 2 days. At the specified time points, the medium was removed and cells were washed twice with PBS, fixed with 4% paraformaldehyde (PFA) for 10 min, and stained for TRAP using a commercial TRAP Kit (Sigma). TRAP-positive cells containing ≥3 nuclei were counted under a light microscope.

### Cell viability

The toxic effect of different concentrations of SrCl_2_ on BMMs was examined by the Cell Counting Kit-8 (CCK-8) following the manufacturer’s instructions. Briefly, BMMs were cultured with different concentrations of SrCl_2_ (0.32, 0.625, 1.25, 2.5, 5, 10 and 20 mM) with and without Ti particles for 48 h. Then 10 μl CCK-8 was added to each well, and the plates were incubated at 37 °C for 3 h. Absorbance at 450 nm was measured using a micro-plate reader (BioTek, Winooski VT, USA) to assess cellular viability.

### Bone resorption assay

BMMs (1 × 10^5^ cells/well) were seeded directly on 24-well Osteo Assay Plate (OAP) (Corning, New York, USA) and treated with RANKL and M-CSF. These cells were incubated with Ti particles or not in the presence or absence of SrCl_2_ (2.5, 5 and 10 mM) for 5 days and then removed with the SB3200 Ultrasonic Generator. The bottom of the well was photographed by inverted microscope (Axiovert 40 CFL; Zeiss, Jena, Germany) and the bone resorption area per well was quantified using photo-editing software (Paint.NET)

### Western blot analysis

Total protein in RAW264.7 cells was isolated at various times. The 20 μg protein samples were used for electrophoresis, and the proteins were then transferred to polyvinylidene fluoride membranes. These membranes were then marked by antibodies specific for phosphor-IκBα, IκBα, phosphor-P65, P65 (all 1:1000; Cell Signaling Technology, Cambridge, MA, USA), and β-actin (1:1000; Novus, Colorado, USA). These proteins were marked by secondary antibodies and then cultured with an eikonogen. The antibody reactivity was detected by exposure in an Odyssey infrared imaging system (LI-COR).

### NF-κB reporter construct and luciferase assay

The NF-κB-responsive reporter NF-κB-Luc (BD Biosciences) was used according to a method previously described^34^. Briefly, reporter plasmids were transfected into RAW264.7 cells (1 × 10^5^ cells/well) using Lipofectamine 2000 reagent (Invitrogen) in α-MEM without FBS and antibiotics. Five hours later, the medium was changed to α-MEM containing 10% FBS, RANKL and Ti particles to stimulate NF-κB activity in the presence of SrCl_2_ or not. Cells were extracted with passive lysis buffer (Promega Corporation, Madison WI) for 24 h, and luciferase activity was measured using the Luciferase Assay System of Promega on a microplate luminometer (Turner Designs, Sunnyvale, CA, USA).

### Enzyme-linked immunosorbent assay (ELISA) analysis

Mouse-specific ELISA kits were used to analyze the amounts of TNF-α, IL-1β and IL-6 produced in the supernatants from BMMs incubated with 30 ng/mL M-CSF, 50 ng/mL RANKL, 0.1 mg/ml Ti particles with/without SrCl_2_ pretreatment to determine relative cytokine levels following the manufacturer’s instruction.

### Experimental animals and drug treatment

As previous described^13^, we established a murine calvarial osteolysis model to investigate the protective effects of SrRan on wear debris-induced bone destruction *in vivo*. Briefly, 60 8-week-old male C57BL/6 mice were randomly divided into four groups: sham control (sham), Ti particles only (vehicle), Ti particles with low SrRan concentration (low-SrRan), and Ti particles with high SrRan concentration (high-SrRan). Animals in the sham group underwent sham surgery only, whereas animals in the vehicle, low- and high-SrRan groups received 30 mg Ti particles. Mice in the low- and high-SrRan groups were gavage-fed with SrRan (450 mg/kg and 900 mg/kg, respectively) every day for 2 weeks. Mice in the sham and vehicle groups received PBS. After 14 days, the mice were sacrificed in a CO_2_ chamber and the calvariae were harvested for micro-computed tomography (μCT), molecular and histological analysis.

### μCT scanning

The calvariae (n = 5 per group) were fixed with 10% formaldehyde for 24 h and analyzed by μCT equipped with a SkyScan1176 scanner and relevant analysis software (SkyScan, Aartselaar, Belgium). The scanning protocol was set at an equidistant resolution of 18 μm with 80 kV and 100 μA energy X-ray. After three-dimensional (3D) image reconstruction, a cylinder-shaped region of interest (ROI; 3 × 3 × 1 mm) around the midline suture was selected for further quantitative analysis. The number of pores, bone mineral density (BMD, mg/mm^2^) and bone volume against tissue volume (BV/TV, %) in each sample were obtained using CT Analyser software (Skyscan).

### Histological and histomorphometric analysis

Calvariae (n = 5 per group) were fixed in 4% PFA for 2 days, decalcified in 10% EDTA for 21 days and embedded in paraffin wax. Cross-sections (5 μm) were cut in the coronal plane on a microtome. Sections were prepared for hematoxylin and eosin (H&E) and TRAP staining using standard procedures. Photos of the stained sections were obtained using an Olympus microscope at a magnification of ×20 with the midline suture at its center. Histomorphometric analysis was carried out on the ROI of four consecutive sections with the help of image analysis software (Image Pro-Plus 6.0, Media Cybernetics, MD, USA). The area eroded bone surface (mm^2^) was determined and quantified using the method established by von Knoch^35^. The periosteum thickness (mm) was used to determined inflammatory cell infiltration as described previously^13^,^36^. The presence of dark-purple-stained granules, accumulated along the eroded bone surface, was considered the specific criterion for TRAP-positive cells. The number of TRAP-positive cells, and the percentage of osteoclast per bone surface (OCs/BS, %), were calculated as previously described^35^,^37^.

For histological detection of p65, RANKL, TNF-α, IL-1β, and IL-6, sections were dewaxed, gradient hydrated, and blocked with 5% hydrogen peroxidase for 5 min. Then, the sections were incubated with primary antibodies against p65 (1:1000), RANKL (1:500), TNF-α (1:200), IL-1β (1:200), and IL-6(1:500) (Abcam, Camb, UK) for 12 h at 4 °C. After washing, sections were incubated with primary secondary antibodies and then avidin-biotin enzyme reagents for 30 min. Rinsed sections were counterstained with hematoxylin.

### RNA isolation and reverse transcription polymerase chain reaction (RT-PCR)

Briefly, total RNA was isolated from calvariae (n = 5 per group) frozen in liquid nitrogen or BMMs using Trizol reagent (Invitrogen, Carlsbad, CA) following the manufacturer’s instruction. 1 μg of total RNA could be used to synthesize first-strand cDNA by reverse-transcription incubated with SuperScript II reverse transcriptase (Invitrogen, Carlsbad, CA). According to the manufacturer’s recommendation, RT-PCR was performed on a DNA synthesizer (Bio-rad). In the study, a housekeeping gene, *glyceraldehydes 3-phosphate dehydrogenase (GAPDH)*, was the internal control gene. The primer sequences used in this study are shown as follows: *cathepsin K (Cath-K)* forward 5′-CTTCCAATACGTGCAGCAGA-3′ and reverse 5′-TCTTCAGGGCTTTCTCGTTC-3′; *calcitonin receptor (CTR)* forward 5′-ACCGACGAGCAACGCCTACGC-3′ and reverse 5′-GCCTTCACAGCCTTCAGGTAC-3′; *matrix metalloproteinase-9 (MMP-9)* forward 5′-CAAAGACCTGAAAACCTCCAA-3′ and reverse 5′-GGTACAAGTATGCCTCTGCCA-3′; *nuclear factor of activated T-cells 1 (NFATc1)* forward 5′-CCGTTGCTTCCAGAAAATAACA-3′ and reverse 5′-TGTGGGATGTGAACTCGGAA-3′; c-Fos forward 5′-CCAGTCAAGAGCATCAGCAA-3′ and reverse 5′-AAGTAGTGCAGCCCGGAGTA-3′; TRAP forward 5′-CTGGAGTGCACGATGCCAGCGACA-3′ and reverse 5′- TCCGTGCTCGGCGATGGACCAGA-3′; *TNF-α* forward 5′-CCACCACGCTCTTCTGTCTAC-3′ and reverse 5′-AGGGTCTGGGCCATAGAACT-3′; *IL-1β* forward 5′-GCCCATCCTCTGTGACTCAT-3′ and reverse 5′-AGGCCACAGGTATTTTGTCG-3′; *IL-6* forward 5′-TTCACAAGTCGGAGGCTT-3′ and reverse 5′-CAGTTTGGTAGCATCCAT-3′; *RANKL* forward 5′- TCCTGAGACTCCATGAAAACG-3′ and reverse 5′- CCCACAATGTGTTGCAGTTC-3′; *GAPDH* forward 5′-ACCCAGAAGACTGTGGATGG-3′ and reverse 5′-CACATTGGGGGTAGGAACAC-3′.

### Statistical analysis

Data were expressed as mean ± standard deviation of triplicate measurements. The significance of differences between groups was assessed by a two-way analysis of variance, including a Student-Newman-Keuls test for post hoc comparisons. A *P* value of  < 0.05 was considered to be significantly different. The software SPSS 11.0 (SPSS Headquarters, Chicago, IL) was used to carry out the statistical computations.

## Additional Information

**How to cite this article**: Zhu, S. *et al*. Strontium inhibits titanium particle-induced osteoclast activation and chronic inflammation via suppression of NF-κB pathway. *Sci. Rep.* **6**, 36251; doi: 10.1038/srep36251 (2016).

**Publisher’s note:** Springer Nature remains neutral with regard to jurisdictional claims in published maps and institutional affiliations.
